# Supplementary material for: Enabling large-scale screening of Barrett’s esophagus using weakly supervised deep learning in histopathology
Source: Nat Commun. 2024 Mar 11;15:2026. doi: 10.1038/s41467-024-46174-2 (PMC10928093; doi:10.1038/s41467-024-46174-2)
Supplement: Supplementary file 1 — Supplementary Information [file 41467_2024_46174_MOESM1_ESM.pdf]

Supplementary Information

Supplementary figures and tables

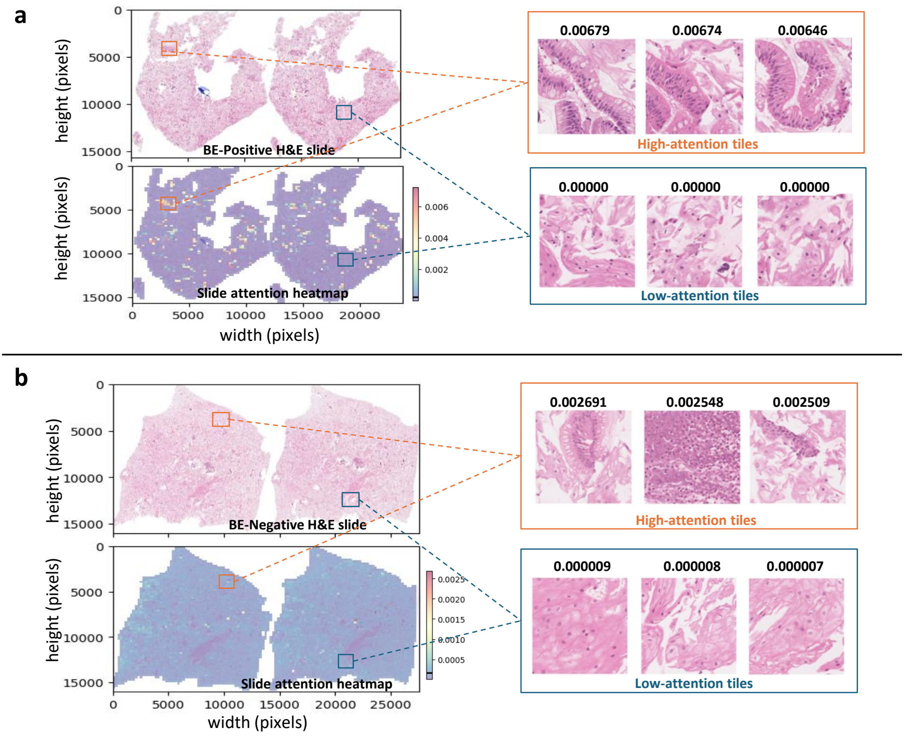

Supplementary Fig. 1: Qualitative analysis of hematoxylin and eosin (H&E) BE-TransMIL model's capability to generalize to out of distribution data. **a**, Goblet cells can be seen in the true positive slide high attention tiles; low attention tiles do not show any goblet cells. **b**, Slide attention heatmap of true negative slide shows nearly uniform attention; high- and low-attention tiles do not have any goblet cells.

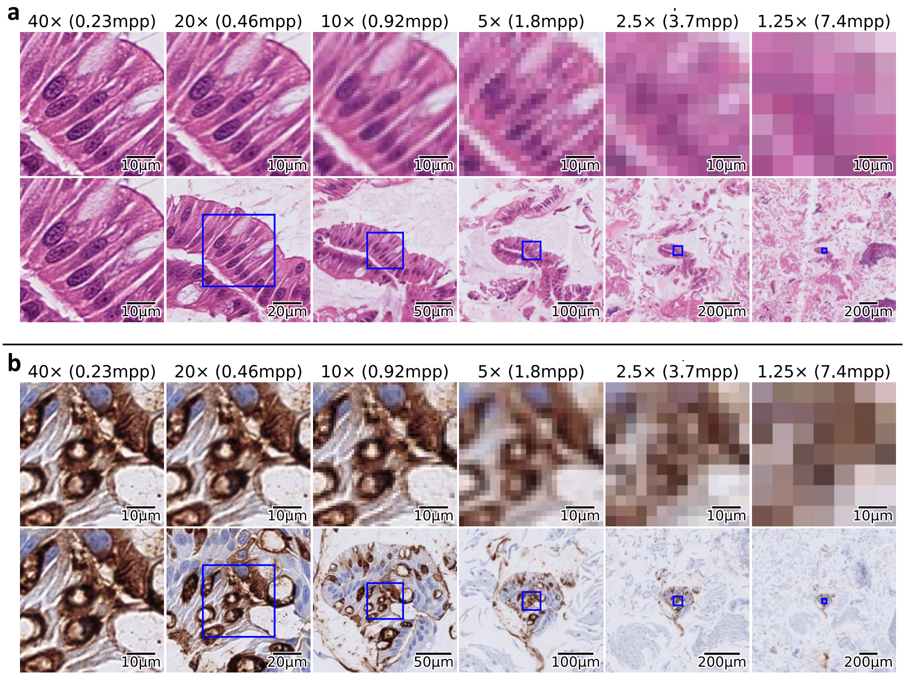

Supplementary Fig. 2: 224×224 tiles of a slide for different objective magnifications: top row shows the same field-of-view at different resolutions, bottom row shows different fields-of-view at different magnifications for a given tile size, with the blue box showing the corresponding region in the first row. **a**, H&E tiles. **b**, trefoil factor 3 (TFF3) tiles.

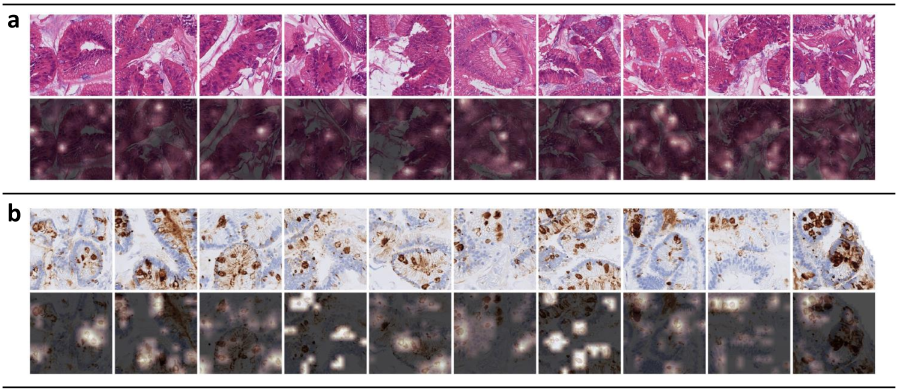

Supplementary Fig. 3: Grad-CAM saliency maps of the top 10 tiles (with highest attention values) of a BE-positive slide (ResNet50, layer 4). **a**, H&E BE-TransMIL model. **b**, TFF3 BE-TransMIL model.

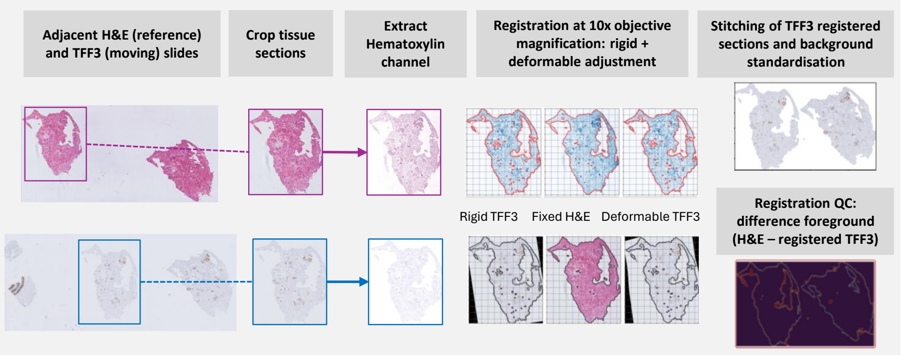

Supplementary Fig. 4: Registration of adjacent TFF3 and H&E slides.

**Encoder**	**AUROC**	**AUPR**	**Accuracy**	**Sensitivity**	**Specificity**
SwinT	0.905 ± 0.018	0.892 ± 0.022	0.836 ± 0.017	0.801 ± 0.045	0.856 ± 0.047
DenseNet121	0.919 ± 0.026	0.907 ± 0.031	0.855 ± 0.025	0.824 ± 0.080	0.874 ± 0.025
ResNet18	0.922 ± 0.011	0.906 ± 0.012	0.849 ± 0.019	**0.833 ± 0.063**	0.858 ± 0.058
**ResNet50**	**0.931 ± 0.021**	**0.919 ± 0.031**	**0.876 ± 0.047**	0.813 ± 0.060	**0.915 ± 0.077**

Supplementary Table 1: H&E BE-TransMIL 4-fold cross-validation performance (0.5 probability threshold) on the discovery validation data splits using different types of image encoders. ResNet50 encoder shows most favorable overall performance.

**Encoder**	**AUROC**	**AUPR**	**Accuracy**	**Sensitivity**	**Specificity**
SwinT	0.967 ± 0.006	0.955 ± 0.007	0.918 ± 0.016	**0.908 ± 0.014**	0.925 ± 0.032
DenseNet121	0.965 ± 0.006	**0.958 ± 0.008**	0.914 ± 0.014	0.867 ± 0.050	**0.943 ± 0.031**
ResNet18	0.963 ± 0.002	0.946 ± 0.014	0.907 ± 0.023	0.890 ± 0.030	0.918 ± 0.055
**ResNet50**	**0.967 ± 0.003**	0.951 ± 0.014	**0.922 ± 0.009**	0.893 ± 0.039	0.939 ± 0.016

Supplementary Table 2: TFF3 BE-TransMIL 4-fold cross-validation performance (0.5 probability threshold) on the discovery validation data splits using different types of image encoders. ResNet50 encoder shows most favorable overall performance, better or consistent with DenseNet121 encoder.

**Mag.**	**AUROC**	**AUPR**	**Accuracy**	**Sensitivity**	**Specificity**
5×	0.941 ± 0.003	0.918 ± 0.010	0.863 ± 0.0147	**0.857 ± 0.048**	0.867 ± 0.052
10×	**0.960 ± 0.006**	**0.954 ± 0.005**	**0.911 ± 0.014**	0.834 ± 0.033	0.958 ± 0.018
20×	0.953 ± 0.009	0.951 ± 0.007	**0.911 ± 0.011**	0.811 ± 0.059	**0.972 ± 0.032**

Supplementary Table 3: H&E BE-TransMIL (ResNet50) replication experiments (mean and standard deviation over n=5 random initializations) at different objective magnifications ('Mag.'): performance (0.5 probability threshold) on a 10% data split from the discovery developmental set. 10× objective magnification shows most favorable overall performance. The variance across initializations is consistently small.

**Encoder**	**AUROC**	**AUPR**	**Accuracy**	**Sensitivity**	**Specificity**
SwinT-FT	0.905 ± 0.018	0.892 ± 0.022	0.836 ± 0.017	**0.801 ± 0.045**	0.856 ± 0.047
CTransPath-PT	0.865 ± 0.021	0.849 ± 0.001	0.8201 ± 0.018	0.718 ± 0.034	0.882 ± 0.003
CTransPath-FT	**0.928 ± 0.018**	**0.916 ± 0.018**	**0.875 ± 0.012**	0.781 ± 0.063	**0.929 ± 0.042**

Supplementary Table 4: H&E BE-TransMIL (SwinT) cross-validation performance (0.5 probability threshold) on the discovery validation data splits using a histopathology pretrained encoder CTransPath [7] compared to vanilla SwinT pretrained on ImageNet. PT stands for pretrained where the encoder is frozen and FT is with end-to-end fine-tuning including the encoder on Cytosponge H&E slides.

Failure type	Total errors	Shared errors	H&E-only errors	TFF3-only errors
False negatives	34 (100%)	19 (55.88%)	13 (38.23%)	2 (6.67%)
False positives	56 (100%)	13 (23.21%)	31 (55.35%)	12 (21.42%)

Supplementary Table 5: Failure quantities computed for H&E and TFF3 BE-TransMIL models. Percentages in parentheses are with respect to the total number of errors for each failure type.

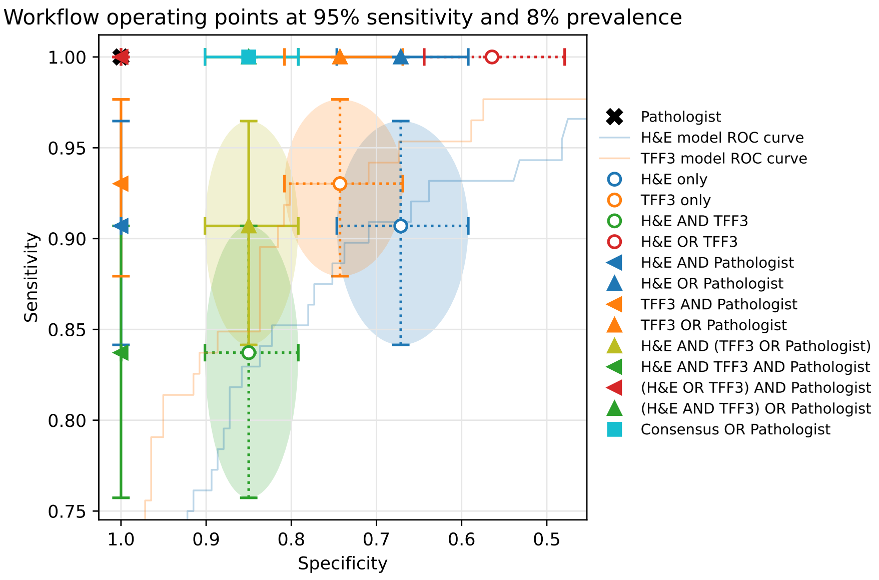

Supplementary Fig. 5: **Performance analysis of multiple ML-assisted workflows.** The sensitivity and specificity of each workflow with respect to pathologist (cross at top-left corner) is presented alongside 95% confidence intervals. ROC curves of the H&E and TFF3 models are also presented.

**Workflow**	**Pathologist review**	**TFF3 staining**	**Obs. prevalence**
Pathologist	100%	100%	8%
H&E only	0%	0%	0%
TFF3 only	0%	100%	0%
H&E AND TFF3	0%	37% [31–45%]	0%
H&E OR TFF3	0%	63% [55–69%]	0%
**H&E AND Pathologist**	**37% [31–45%]**	**37% [31–45%]**	**19% [16–24%]**
H&E OR Pathologist	63% [55–69%]	63% [55–69%]	1% [1–2%]
TFF3 AND Pathologist	31% [25–38%]	100%	24% [20–31%]
TFF3 OR Pathologist	69% [62–75%]	100%	1% [0–2%]
H&E AND (TFF3 OR Path.)	17% [12–23%]	37% [31–45%]	3% [1–7%]
H&E AND TFF3 AND Path.	20% [16–26%]	37% [31–45%]	33% [26–43%]
**(H&E OR TFF3) AND Path.**	**48% [41–55%]**	**100%**	**17% [14–20%]**
(H&E AND TFF3) OR Path.	80% [74–84%]	100%	2% [1–2%]
Consensus OR Pathologist	28% [21–35%]	100%	5% [2–8%]

Supplementary Table 6: Pathologists' workload as a fraction of current reviewed cases, TFF3 staining as a fraction of the current cases, and observed (obs.) prevalence of Barrett's esophagus (BE) for possible workflows using the BE-TransMIL models, along with their 95% confidence intervals (CIs).

Patient demographics

Supplementary Table 7: Patient demographics for discovery and external evaluation datasets.

**Patient demographics for discovery (DELTA) dataset**	
Age	Median 66 years, IQR 58-74 years.
Sex	Male: n=726 (57.7%)<br>Female: n=508 (40.3%)<br>Missing: n=25 (2%).
Ethnicity	Not provided.
**Patient demographics for external (BEST2) dataset**	
Age	Median 63 years, IQR 52-70 years.
Sex	Male: n=439 (60.5%)<br>Female: n=241 (33.2%)<br>Missing: n=45 (6.2%).
Ethnicity	Not provided.

Summary of key study elements

In this section, we summarize the key study elements of our paper listed according to recent reporting guidelines [9, 10] for applications of machine learning (ML) in clinical research. We present the study setup (Supplementary Table 8), model details (Supplementary Table 9), experimental details (Supplementary Table 10) and reproducibility (Supplementary table 11).

Supplementary Table 8: Study setup

**Study design**	
Clinical question	Can we detect BE from the routinely-stained H&E slides using weakly-supervised deep learning methods?
Model task and outputs	Binary classification of H&E whole-slide images into BE positive or negative. Slide attention heatmaps showing high and low attended tiles by the model to make the predictions.
Intended use of results/target user	Predictions and model outputs (e.g., slide attention heatmaps) can be made available to clinical supporters as part of the Cytosponge-TFF3 test management, for instance, to assist pathologists in semi-automated workflows (see Results), enhance the scalability of the test, and improve patient outcomes.
**Study population and setting**	
Population	Comprises of data from patients attending Barrett's surveillance or screening programs as enrolled in one of the two studies, namely, DELTA and BEST2 (see Methods: Discovery and external evaluation datasets).
Study setting	DELTA implementation study and BEST2 clinical trials registered in the UK (see Methods: Discovery and external evaluation datasets).
Data source	Cyted Ltd, Cambridge, UK.
Cohort selection (exclusion and inclusion criteria)	All available slides from the DELTA study.<br>Licensed slides available to Cyted Ltd from the BEST2 study.
**Data sources**	
Data types	Whole-slide images in NDPI format (discovery dataset) and SVS format (external dataset), converted into TIFF at objective magnification 10× (0.92 μm/pixel). Tiles generated of size 224 × 224 pixels ($\approx 200 \times 200\,\mu m$) from whole-slide images of sizes of order of 1×10^8 pixels. Structured metadata in TSV format.
Data collection and processing	See Methods: Discovery and external evaluation datasets, Data preprocessing.
Data structures	RGB image (array of 3 channels), binary label for BE.
Data partitions	See Table 1.

Supplementary Table 9: Model details

**Model architecture**	
ML method and rationale	Weakly supervised multiple instance learning (MIL) method can achieve a high diagnostic performance to detect BE from H&E slides due to a strong alignment with the nature of the task: a slide is labeled BE positive when goblet cells are detected in a small area of the whole-slide image — this is a classical MIL task and resembles the assessment criteria of expert histopathologists. We use a weakly supervised deep learning network architecture inspired by Transformer-MIL proposed in [8]. The resulting model architecture is called BE-TransMIL. Benchmarked encoders: ResNet18, ResNet50, DenseNet121, Swin-T (see Methods: Model architecture).
Features	Learnable features selected by the deep learning model. Interpretability analysis highlights the following features with higher attention values given by the model (see Results, Fig. 2, Fig. 3, Fig. 5). H&E slides: Mucin-containing goblet cells are visible with a distinct cellular morphology in the high-attention tiles. TFF3 slides: Goblet cells show positive staining of the brown histochemical stain in the high-attention tiles.
**Model training**	
Hardware, software, packages	- Azure ML infrastructure (https://azure.microsoft.com/) for pre-processing TFF3 slides, training and evaluating deep learning models.<br>- HistoQC configv2.1 [5] for pre-processing H&E slides.<br>- MONAI 1.2.dev2310 [6] for data pre-processing and tiling-on-the-fly.<br>- Eight NVIDIA V100 GPUs for training, one NVIDIA V100 GPU for inference.<br>- 40 CPU cores for tiling on-the-fly.<br>- Python 3.9 for code implementation.<br>- PyTorch 1.1.0 [3] and PyTorch-Lightening1.6.5 [4] for implementing the deep learning models and model evaluation.<br>- Scikit-learn 1.2.2 [2], Scikit-image 0.19.3 [11] for data processing and analysis.<br>- SimpleITK 2.1.1.2 [1] for registration of H&E and TFF3 slides.
Hyper-parameters	BE-TransMIL models trained for binary classification task using BCE loss, ADAM optimizer ($\beta_1 = 0.9, \beta_2 = 0.99$), batch size 8, varying bag size (ResNet18: 2300, ResNet50: 1200, Swin-T: 1100, DenseNet121: 700), hidden dimension of attention pooling layer 2048, 50 epochs, learning rate 3e-5, weight decay 0.1, random seed 42. Hyperparameter tuning of models based on classification accuracy, prioritized on specificity (in order to identify negative cases with high confidence for screening populations).
Scalability methods	activation checkpointing (training), encoding in chunks (whole slide inference). See Methods: Model description for details.

Supplementary Table 10: Experimental details

**Evaluation setup**	
Data labels (Gold standard)	The BE positive or negative labels are manually extracted from pathologists' diagnostic reports. Pathologists visually assessed each slide pair (H&E, TFF3) to make their BE diagnosis.
Missingness	Not applicable
Data split	80:20 split of discovery dataset into development (training, validation) and test datasets (See Fig 1c). Four-fold cross validation leading to 60:20:20 split over the entire discovery dataset. Validation and test datasets were randomly selected, stratified according to distributions of class labels and patient pathway (surveillance or screening).
Evaluation measures (metrics)	AUROC, AUPR, Accuracy, Sensitivity, Specificity (at threshold=0.5) for comparing and model selection, with priority to AUROC and AUPR as these are threshold-agnostic metrics.<br>Accuracy, AUROC, AUPR, Sensitivity, Specificity at selected operating point (corresponding to 0.85 validation sensitivity for clinical utility) for reporting performance on discovery and external test sets.<br>ROC curves with bootstrapping for 95% CI.
**Model validation**	
Internal model validation	Discovery dataset comprises of cases from the DELTA implementation study.<br>For each fold in four-fold cross-validation, discovery training set consists of 912 slides and validation set of 229 slides for model selection and comparison.<br>Discovery test set consists of 229 slides for model evaluation.
External model validation	External dataset comprises of slide images from the BEST2 case-control clinical trial. The external validation set consists of 725 slides.
Interpretability analysis	We analyze the attentions of the model qualitatively and quantitatively, correlate the findings with TFF3 stain for which goblet cells show positive staining, and analyze failure modes to ensure model outputs are interpretable (See Results). The analysis includes 1) Visual assessment of slide attention heatmaps and top/bottom attention tiles to analyze the visual features selected by the models to make a decision. 2) GradCAM saliency maps to analyze fine-grained attention in tiles. 3) Stain-attention correspondence analysis to correlate the attentions with TFF3 stain ratio. 4) Failure modes analysis to assess the failures of shared and individual mistakes of H&E and TFF3 models.

Supplementary Table 11: Transparency, reproducibility, code re-use

Data availability	Data cannot be shared by corresponding author due to license agreements of Cyted Ltd with partners. The study protocols for DELTA and BEST2 are publicly available. All data used was deidentified. The dataset is governed by data usage policies specified by the data controller (University of Cambridge, Cancer Research UK). We are committed to complying with Cancer Research UK's Data Sharing and Preservation Policy. Whole-slide images used in this study will be available for non-commercial research purposes upon approval by a Data Access Committee according to institutional requirements. Applications for data access should be directed to rcf29@cam.ac.uk.
Code availability	All the code associated with the paper is open-sourced and available for public use[1]. The main repository, BE-TransMIL, can be found at https://github.com/microsoft/be-trans-mil. It provides code for data processing and result analysis. It includes Microsoft Health Intelligence Machine Learning toolbox (hi-ml) https://github.com/microsoft/hi-ml as a submodule, which contains code and library requirements for data preprocessing, network architectures, and training and evaluation of weakly supervised deep learning models for computational pathology (https://github.com/microsoft/hi-ml/tree/main/hi-ml-cpath#readme). Detailed instructions on using the hi-ml software are provided at https://github.com/microsoft/hi-ml/blob/main/docs/source/histopathology.md

References

[1] Bradley Lowekamp, David Chen, Luis Ibáñez, and Daniel Blezek. The Design of SimpleITK. *Frontiers in Neuroinformatics*, 7:2013. https://www.frontiersin.org/articles/10.3389/fninf.2013.00045 doi: 10.3389/fninf.2013.00045 ISSN: 1662-5196

[2] F. Pedregosa, G. Varoquaux, A. Gramfort, V. Michel, B. Thirion, O. Grisel, M. Blondel, P. Prettenhofer, R. Weiss, V. Dubourg, J. Vanderplas, A. Passos, D. Cournapeau, M. Brucher, M. Perrot, and E. Duchesnay. Scikit-learn: Machine Learning in Python. *Journal of Machine Learning Research*, 12:2825–2830, 2011.

[3] A. Paszke, S. Gross, F. Massa, A. Lerer, J. Bradbury, G. Chanan, T. Killeen, Z. Lin, N. Gimelshein, L. Antiga, A. Desmaison, A. Kopf, E.

[1] The software described in the repository is provided for research and development use only. The software is not intended for use in clinical decision-making or for any other clinical use, and the performance of model for clinical use has not been established. You bear sole responsibility for any use of this software, including incorporation into any product intended for clinical use.

Yang, Z. DeVito, M. Raison, A. Tejani, S. Chilamkurthy, B. Steiner, L. Fang, J. Bai, and S. Chintala. PyTorch: An Imperative Style, High-Performance Deep Learning Library. *Advances in Neural Information Processing Systems*, 32:8024–8035, 2019.

[4] W.A. Falcon, A. Bojarski, S. Beery, and W. Zaremba. PyTorch Lightning. *arXiv preprint arXiv:2012.09184*, 2020.

[5] Andrew Janowczyk, Ren Zuo, Hannah Gilmore, Michael Feldman, and Anant Madabhushi. HistoQC: An Open-Source Quality Control Tool for Digital Pathology Slides. *JCO Clinical Cancer Informatics*, (3):1–7, 2019. doi: 10.1200/CCI.18.00157

[6] M. Jorge Cardoso, Wenqi Li, Richard Brown, Nic Ma, Eric Kerfoot, Yiheng Wang, Benjamin Murrey, Andriy Myronenko, Can Zhao, Dong Yang, Vishwesh Nath, Yufan He, Ziyue Xu, Ali Hatamizadeh, Andriy Myronenko, Wentao Zhu, Yun Liu, Mingxin Zheng, Yucheng Tang, Isaac Yang, Michael Zephyr, Behrooz Hashemian, Sachidanand Alle, Mohammad Zalbagi Darestani, Charlie Budd, Marc Modat, Tom Vercauteren, Guotai Wang, Yiwen Li, Yipeng Hu, Yunguan Fu, Benjamin Gorman, Hans Johnson, Brad Genereaux, Barbaros S. Erdal, Vikash Gupta, Andres Diaz-Pinto, Andre Dourson, Lena Maier-Hein, Paul F. Jaeger, Michael Baumgartner, Jayashree Kalpathy-Cramer, Mona Flores, Justin Kirby, Lee A. D. Cooper, Holger R. Roth, Daguang Xu, David Bericat, Ralf Floca, S. Kevin Zhou, Haris Shuaib, Keyvan Farahani, Klaus H. Maier-Hein, Stephen Aylward, Prerna Dogra, Sebastien Ourselin, and Andrew Feng. MONAI: An open-source framework for deep learning in healthcare. *arXiv preprint arXiv:2211.02701*, 2022.

[7] Xiyue Wang, Sen Yang, Jun Zhang, Minghui Wang, Jing Zhang, Wei Yang, Junzhou Huang, and Xiao Han. Transformer-based unsupervised contrastive learning for histopathological image classification. *Medical Image Analysis*, 81:102559, 2022. doi: 10.1016/j.media.2022.102559 url: https://www.sciencedirect.com/science/article/pii/S1361841522002043

[8] Andriy Myronenko, Ziyue Xu, Dong Yang, Holger R. Roth, and Daguang Xu. Accounting for dependencies in deep learning based multiple instance learning for whole slide imaging. In *Medical Image Computing and Computer Assisted Intervention – MICCAI 2021*, pages 329–338, Cham, 2021. Springer. doi: 10.1007/978-3-030-87237-3_32

[9] Laura M. Stevens, Bobak J. Mortazavi, Rahul C. Deo, Lesley Curtis, and David P. Kao. Recommendations for reporting machine learning analyses in clinical research. *Circulation: Cardiovascular Quality and Outcomes*, 13(10):e006556, 2020. Publisher: Am Heart Assoc.

[10] Tina Hernandez-Boussard, Selen Bozkurt, John PA Ioannidis, and Nigam H. Shah. MINIMAR (MINimum Information for Medical AI Reporting): developing reporting standards for artificial intelligence in health care. *Journal of the American Medical Informatics Association*, 27(12):2011–2015, 2020. Publisher: Oxford University Press.

[11] Stéfan van der Walt, Johannes L. Schönberger, Juan Nunez-Iglesias, François Boulogne, Joshua D. Warner, Neil Yager, Emmanuelle Gouillart, Tony Yu, and the scikit-image contributors. scikit-image: image processing in Python. *PeerJ*, 2:e453, 2014. doi: 10.7717/peerj.453
